# Children’s inequality aversion in intergroup contexts: The role of parents’ social dominance orientation, right-wing authoritarianism and moral foundations

**DOI:** 10.1371/journal.pone.0261603

**Published:** 2021-12-31

**Authors:** Margherita Guidetti, Luciana Carraro, Luigi Castelli

**Affiliations:** Dipartimento di Psicologia dello Sviluppo e della Socializzazione, Università di Padova, Padova, Italy; National Taiwan University, TAIWAN

## Abstract

Although children are overall sensitive to inequality and prefer fair allocation of resources, they also often display ingroup favouritism. Inquiring about the factors that can shape the tension between these two driving forces in children, we focused on the role of parents. Extending the limited literature in this field, the present work examined whether individual differences in 3-to 11-year-old White children’s (N = 154, 78 boys) evaluations of fair versus pro-ingroup behaviours in an intergroup context vary as a function of both mothers’ and fathers’ social dominance orientation (SDO), right-wing authoritarianism (RWA), and moral foundations. Parents completed a questionnaire. Children were presented with a scenario in which two ingroup members distributed candies to two other children, one White and one Black, either in an egalitarian way or displaying a clear ingroup favouritism. Afterwards, their attitudes towards the two ingroup members who had distributed the candies were assessed through both an Implicit Association Test and explicit questions. Although children displayed on average an explicit preference for the fair over the pro-ingroup target, this preference did not emerge at the implicit level. Most importantly, both children’s explicit and implicit attitudes were related to mothers’ SDO, indicating that at increasing level of mothers’ SDO children’s inequality aversion tended to drop. Overall, these results emphasize the relevance of mothers’ support for social hierarchy in relation to the way in which children balance the two competing drives of equality endorsement and pro-ingroup bias.

## Introduction

Previous research has clearly shown two overall tendencies in the way children appraise distributive behaviours. On the one hand, children are sensitive to the relative amount of resources that the various recipients obtain, strongly preferring a fair allocation of resources [e.g., 1, 2]. This phenomenon reflects a general inequality aversion. On the other hand, it further appears that children do also consider the group membership of the recipients, so that to favor their ingroup at the expenses of pure egalitarian behaviors (i.e., parochialism) [e.g., 1, 3]. Therefore, in intergroup contexts there is often a tension between two driving forces: A norm towards egalitarianism and a bias in favour of one’s own social group. This applies both in relation to the way children personally distribute resources and in relations to the evaluation of peers who are involved in the distribution of resources in intergroup settings. In the present work, we will focus on the latter aspect and, more specifically, on how the relative evaluation of ingroup peers who behave in an egalitarian versus pro-ingroup way is related to mothers’ and fathers’ authoritarian dispositions and moral foundations.

### Children’s inequality aversion and ingroup bias

Children are sensitive to social inequality since a very young age. Indeed, infants as young as 12 months have been shown to more strongly value fair behaviours in comparison with unequal distributions of resources [4–6]. This has been interpreted as a human innate tendency towards equality and prosocial behaviors [7], likely pointing to the innate foundations of some aspects of human morality [8].

While preverbal children’s preferences are usually inferred from looking times and manual choices [e.g., 4], older children (i.e., from about 3–4 years of age) are most often required to either directly distribute resources or to evaluate other individuals as a function of the way they had been distributing resources. Even though there is an overall preference for fair behaviors, several factors can actually moderate this tendency. Indeed, in resource allocation tasks, children’s self-interest (i.e., the maximization of one’s own gain) may play a crucial role, although the attainment of a personal advantage tends to significantly decline with age [1, 9–11]. This is at least partially due to an increased concern about appearing fair to external observers [12]. In a similar vein, children are more willing to share when the recipient is a sibling or a friend rather than an acquaintance or a stranger [13–15], and to equally divide rewards obtained by working collaboratively with a partner, even if they could be easily monopolized [16, 17]. Importantly, the enforcement of a fairness norm can also be significantly influenced by the group membership of the various actors. Indeed, children soon start to display an ingroup bias, namely a favouritism for one’s own group, in both pre-existing [18–24] and experimentally-created [25–27] intergroup settings.

(Un)fair distribution and (in)justice can be experienced not only when assuming the role of the distributor (e.g., when allocating resources to oneself and another person, or to two other individuals), but also as either the receiver or the observer of the distributive behaviours performed by others. The study of this latter situation, which implies to judge resource allocation between third-parties, is particularly useful in that it allows to remove the potential effects of self-interest. Research findings show that observers enforce fairness norms and are even willing to incur into personal costs in order to punish those who had behaved selfishly, thus creating inequality conditions that had to be somehow restored [i.e., third-party punishment; 28–30].

In a relevant study, Jordan and colleagues [31] employed a third-party punishment task with 6-to 8-year-old children who were given the chance to punish the unfair behaviour of ingroup and outgroup members towards other ingroup and outgroup members. Findings showed that punishment was stronger when the unfair behaviour was perpetrated by an outgroup member and, most importantly, when unfair allocations represented a disadvantage for participants’ own group [see also 32]. These effects are complemented by findings showing that children actually expect that peers would behave in ways that ultimately benefit the ingroup. This indicates that even young children are sensitive to the interplay between a general equality norm and a pro-ingroup norm [3].

### The antecedents of inequality aversion

Although the impact of respondents’ group membership on the reactions towards observed unfairness in intergroup contexts appears to be well-established, less is known about the factors that can shape these effects among children. Research with adult populations indicates that there is a significant inter-individual variability in the way people either condemn or accept social inequality. In particular, high levels of Social Dominance Orientation [SDO; 33] are a significant predictor of people’s tendency to consider inequality as an ordinary and acceptable aspect of social life, framed as a competitive jungle in which personal and group interests should be pursued [34, 35]. Intergroup discrimination and prejudice could thus be promoted or, at least, tolerated, especially in relation to subordinate groups [36, 37]. In sharp contrast, individuals who score low on SDO embrace strikingly different worldview beliefs revolving around the concepts of egalitarianism and fairness.

An emerging field of research has suggested that differences related to political orientation can be detected very early in life [38, 39] and parents’ ideology, through both genes and socialization, may affect their children’s ideology precursors [40–44]. In other words, children of conservative parents seem to differ from children of liberal parents, displaying a set of characteristics predisposing either to conservatism or to liberalism. Differences involve neurocognitive responses to conflict [40], epistemic, existential, and relational needs [41], deference to convention and authority [42] and sensitivity to intergroup fairness violation [43]. It can thus be expected that the social and family environment in which children are socialized might affect their early reactions towards intergroup discrimination as well as, more specifically, their level of inequality aversion when unfairness provides an advantage to the ingroup. So far, only one study has specifically addressed this issue focusing on the potential role of parents [43]. Reifen Tagar and colleagues [43] exposed preschool-aged children to three different between-participants conditions in the context of a minimal group paradigm. In an “unfair-ingroup” condition, children saw a video in which an ingroup member behaved in a selfish way keeping a resource for herself while refusing to share it with an outgroup member. In a “fair-outgroup” condition, children saw an outgroup member who actually split the resource in equal parts between herself and a member of participants’ own group. In a final control condition, participants saw a third-part, whose category membership was not specified, splitting the resource in equal parts between an ingroup and an outgroup member. All children were then involved in a resource allocation task in which they could distribute resources to the ingroup and outgroup agents they had seen before in the videos. Findings showed that parents’ SDO predicted children’s resources allocation to the ingroup member in the “unfair-ingroup” condition, namely when there was a tension between the norms of fairness and ingroup favouritism. Indeed, children of parents low in SDO showed the strongest inequality aversion penalizing the ingroup member who had behaved unfairly, whereas children of parents high in SDO did not penalize such ingroup member. Importantly, parents’ authoritarian predisposition [45, 46] yielded no significant effect.

The equal treatment of other individuals has also clear moral implications. Although the ethics of justice [47] and the care for the weakest [48] have been considered for long time the only forms of morality, the moral foundation theory (MFT) [49–51] argues that the moral domain is more articulated and based upon at least five foundations: in addition to harm/care and fairness/reciprocity (also called the individualizing foundations), ingroup/loyalty, authority/respect and purity/sanctity (the binding foundations) can be included. The MFT, however, has been criticized on several grounds and, most relevant for the aims of the present work, it has been suggested that binding foundations merely reflect resistance to change and right-wing authoritarianism (RWA), whereas individualizing foundations would reflect SDO and opposition to equality [52–55; but see 56]. Accordingly, moral intuitions would not be a primary cause of political ideology, but they should be better conceived as a further outcome of motivated social cognition [57, 58]. Within this overall framework, it thus becomes important to explore whether parents’ moral foundations can end to be further relevant predictors of their children’s inequality aversion.

### The present study

The major goal of the present work is to conceptually replicate and extend the research by Reifen Tagar and colleagues [43]. First, Reifen Tagar and colleagues [43] did not specifically address the potential differential role of mothers and fathers. To this end, we aimed to involve both the mother and the father of each child participant. Previous research has shown that although mothers’ intergroup attitudes, as compared to fathers’ attitudes, are sometimes a better predictor of children’s intergroup attitudes [24, 59], the pattern does not appear to consistently emerge [e.g., 60; see also 61 in relation to the parent–adolescent concordance in authoritarian dispositions]. We thus explored for the first time whether children’s inequality aversion in intergroup settings is differentially related to the psychological dimensions of SDO and authoritarian predisposition of both parental figures.

Two additional key differences concern the kind of groups examined and the way unfair behaviours were presented to children. First, we focused on meaningful and pre-existing groups (i.e., based on skin colour) rather than on minimal groups. As for the second point, in the study by Reifen Tagar and colleagues [43] the unfair ingroup member could be motivated by self-interest (i.e., keeping the resources for herself) and not only by a concern about the welfare of the ingroup. Thus, the relation with parents’ SDO might reflect individual differences in the preference for high status targets, namely for individuals who end up to have more resources as compared to other individuals. In order to more directly assess intergroup dynamics, in the present work participants were asked to evaluate two ingroup members who distributed resources in an equal versus unequal way to an ingroup and outgroup member without gaining any personal benefit from their distribution choices. As said, we thus aimed to more specifically address children’s individual differences in inequality aversion in an intergroup context and how this might be related to parents’ ideological attitudes (i.e., SDO and RWA).

Moreover, we measured children participants’ explicit attitudes in a multifaceted way as well as their implicit attitudes. Indeed, children are early aware about the relevance of fairness norms and may thus respond in a socially desirable way to explicit questions. The use of an implicit measure enabled to assess whether children inequality aversion and parents’ influence are limited to responses that could–at least potentially–be strategically controlled, or they do extend to the more spontaneous appraisal of the fair and pro-ingroup targets.

As for parents’ measures, in addition to assessing parents’ SDO, we employed a well-established measure of Right-Wing Authoritarianism [RWA; 62] in order to further test whether parents’ authoritarianism is indeed a non-significant predictor of children’s inequality aversion. In an exploratory way, we also examined the potential role of parents’ moral foundations [63]. Indeed, children’s judgments about equal and unequal behaviours are deeply related to their moral reasoning [64], and this is especially true as children grow older [65].

Based on research suggesting that moral foundations merely reflect political ideology, rather than the reverse [52–55, 57, 58], we explored whether a specific pattern linking parents’ individualizing and binding moral foundations and children’s responses could be identified or, alternatively, parents’ social attitudes associated to RWA and SDO represent more powerful predictive constructs. This will allow to provide further insights about the possible redundancy of moral foundations with respect to SDO and RWA or, alternatively, to eventually highlight their differential contribution in the prediction of children’s inequality aversion in intergroup settings.

## Method

### Participants

One-hundred and sixty-two families were recruited in one kindergarten and two primary schools in Northern Italy. We tried to enrol as many participants as we could within the schools that collaborated to the project, aiming to achieve at least the same sample size as in the work by Reifen Tagar and colleagues [43]. Critically, we employed a within-participants design (i.e., children evaluated both a fair and pro-ingroup target), as compared to the between-participants design adopted by Tagar and colleagues [43]. This considerably increases statistical power (see [66, 67]). Because our manipulation concerned race, data from 8 non-White children were not included in the analyses and therefore the final sample consisted of 154 children and at least one of their parents. Children were all Italians, except two of them who were from East Europe; all participants were very fluent in Italian. Children were 3-to 11-year-old (*M* = 8.00, *SD* = 2.02) and 78 were males. They were individually interviewed at school in a quiet room after having obtained written consent from the parents, in accordance with the Declaration of Helsinki. The study has been approved in written form by the Ethical Committee for the Psychological Research of the University of Padova, protocol n. 2823. Parents had to complete a paper-and-pencil questionnaire. Questionnaires were returned by 142 mothers (aged *M* = 41.59, *SD* = 4.44) and 128 fathers (aged *M* = 44.41, *SD* = 4.69).

### Procedure

**Fig 1:**
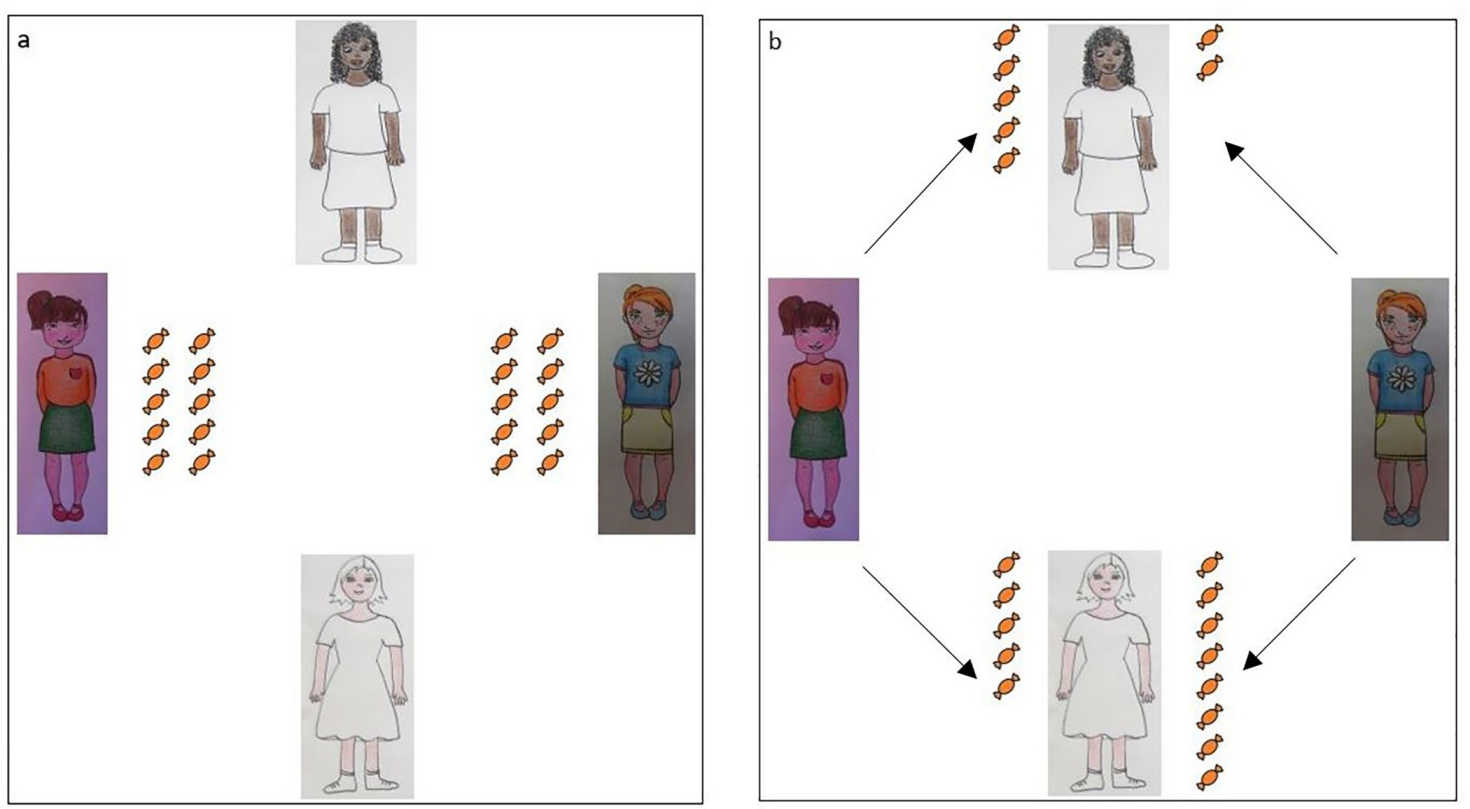
Scenario for female participants. a) Flavia and Claudia have 10 candies each, b) Flavia decides to give 5 candies to this girl (on the top) and 5 candies to this other girl (on the bottom); Claudia decides to give 8 candies to this girl (on the bottom) and 2 candies to this other girl (on the top). *Note*: *The relative order of presentation of the two protagonists*, *the relative position of the two protagonists distributing the candies (i*.*e*., *on the right or left) and the two characters receiving the candies*, *and the association between the behaviours and the protagonists’ names/drawings were counterbalanced across participants*.

All the children participating in the study were presented with a scenario in which two ingroup members (i.e., two White children) distributed candies to two other children, one White and one Black. The experimenter never mentioned the racial membership of the various characters. The four children in the scenario were represented on four distinct drawing cards that were displayed on a table in front of the participant (see Fig 1; cfr. [32] for a similar procedure). The gender of all the portrayed children always matched the gender of the participant. One of the protagonists distributed the candies in an egalitarian way towards the White and Black targets. The behaviour of this “fair child” was described as follows: “The name of this child is Flavio/a. Flavio/a has 10 candies and he/she decides to give 5 candies to this child and 5 candies to this other child”. The experimenter placed 5 real candies near the drawing of the ingroup member (i.e., the White character) and 5 candies near the drawing of the outgroup member (i.e., the Black character). Afterward, the experimenter introduced the other protagonist distributing the candies who, in contrast, displayed a clear ingroup favouritism. The behaviour of this “pro-ingroup” child was described as follows: “The name of this child is Claudio/a. Claudio/a has 10 candies and he/she decides to give 8 candies to this child and 2 candies to this other child”. The experimenter placed 8 candies near the drawing of the ingroup member (i.e., the White character) and 2 candies near the drawing of the outgroup member (i.e., the Black character).

After presenting the scenario, participants sat in front of a computer laptop to perform an Implicit Association Test (IAT; [68]), aimed at assessing the implicit attitudes towards Flavio/a and Claudio/a. Subsequently, children were asked to provide an explanation about the observed behaviours; this open ended question was not analysed in the present work. Afterwards, participants were asked a series of questions aimed at assessing their explicit attitudes towards each of two characters. The order of administration of the questions about the fair and pro-ingroup child was counterbalanced across participants. Finally, they were asked to directly compare the two protagonists.

Parents received and returned a paper-and-pencil questionnaire through teachers. Questionnaires were delivered in closed envelopes and an alphanumeric code allowed child-parent matching. All measures administered to children and parents are detailed below. Parents’ questionnaire, children’s interview, IAT stimuli and data are available at https://osf.io/27pcm/?view_only=fcc39b20653d427d88c8ab8f9aea87c8.

### Children’s measures

#### Implicit association test

A child-friendly IAT [69] measured children’s implicit attitudes towards the two protagonists of the scenarios, in a sequence of five blocks (three practice and two critical blocks) whereby they categorized happy versus sad emoticons [see 41] and images of Claudio/a and Flavio/a (i.e., the two characters who had distributed the candies). To simplify the task, we used a keyboard with two different coloured labels on the two response keys (blue for the left key and red for the right key). Each block included instructions about the category discriminations and key assignments. The instructions were explained to the participants by the experimenter who proceeded only after having assessed that participants had actually understood them.

In the first 10-trial practice block, participants categorized attribute stimuli as happy versus sad emoticons. In the following 20-trial practice block they had to categorize images of Claudio/a and Flavio/a. The third 40-trial critical block required a combined categorization task, and all stimuli were presented (i.e., emoticons and images of the two protagonists). A specific response key had to be pressed if either positive emoticons (i.e., happy) or images of Claudio/a appeared on the screen, whereas another response key had to be pressed if negative emoticons (i.e., sad or angry) or images of Flavio/a were displayed (i.e., compatible block for participants with a more positive attitude towards Claudio/a than towards Flavio/a). In the fourth 20-trial practice block, the response keys for pictures of Claudio/a and Flavio/a were reversed as compared to the second block. The final 40-trial critical block was again a combined categorization task, but this time positive emoticons and images of Flavio/a, on the one hand, and negative emoticons and images of Claudio/a, on the other, shared the same response key (i.e., non-compatible block for participants with a preference for Claudio/a versus Flavio/a). The order of the third and fifth critical block, as well as the order of the second and the fourth block, were counterbalanced across participants, so that half of them performed the compatible block prior to the non-compatible block, as reported above, whereas the other half performed the non-compatible block prior to the compatible block. The happy versus sad emoticons categories included 6 stimuli each, and the target categories included 3 images of Claudio/a and 3 images of Flavio/a.

We computed an IAT score according to the algorithm proposed by Greenwald et al. [70] in such a way that positive values indicated an implicit preference for the fair versus pro-ingroup child. The reliability of the IAT (α = .72) was acceptable and comparable to that observed in previous studies with both adult and children populations [e.g., 41, 69]. Scores on the IAT have proved to be valid predictors of further relevant social behaviours in both adults and children [71, 72].

#### Fair and pro-ingroup behaviour evaluation

Participants were asked to evaluate the behaviour of each child on a 4-point scale (i.e., “Please tell me how this child has behaved according to you”; 1 = *very badly*, 2 = *badly*, 3 = *well*, 4 = *very well*).

#### Intention to interact with the fair and pro-ingroup child

Participants were then asked to report how much they would have liked to play with each protagonist (1 = *not at all*, 2 = *a bit*, 3 = *somewhat*, 4 = *very much*).

#### Traits evaluation of the fair and pro-ingroup child

Participants were asked how much they considered each protagonist to be dirty, kind, bad, good, rude, and clean (1 = *not at all*, 2 = *a bit*, 3 = *somewhat*, 4 = *very much*). After rescaling negative traits, a mean score was computed for both children, α = .58 for the fair, α = .86 for the pro-ingroup child.

#### Preference as a playmate

In the last part of the interview, a direct comparison was proposed. Children had to indicate with whom they would have liked to play the most (4-point response scale: definitely/somewhat with either one or the other protagonist).

#### Traits comparison

Finally, participants were asked to attribute each of the same adjectives used before (i.e., dirty, kind, bad, good, rude, and clean) either to one protagonist, to the other, to both of them or to none of them. We subtracted the number of negative traits from the number of positive traits attributed to each protagonist.

For all the explicit measures described above (except for preference as a playmate), a difference score between the fair and the pro-ingroup protagonist scores was computed in such a way that positive values indicate a preference for the fair as compared to the pro-ingroup child.

### Parents’ measures

After having reported their age and gender, parents completed the Italian short version [73] (8 items) of the Social Dominance Orientation Scale [33] and the Italian version [74] of the Right-Wing Authoritarianism Scale by Funke [62] (12 items). Responses were provided on 7-point Likert scales ranging from *completely disagree* to *completely agree*. After rescaling the appropriate items, we computed average scores for each measure for both mothers (SDO α = .77; RWA α = .62) and fathers (SDO α = .66; RWA α = .71).

Parents also filled in the Italian version [75] of the Moral Foundations Questionnaire [76] (6 items for each of the 5 foundations). In the first part of this questionnaire, participants were asked how relevant a series of considerations are when deciding whether something is right or wrong; in the second part they were asked to indicate their level of disagreement/agreement with a series of sentences. Responses were given on two 6-point scales ranging from *not at all relevant* to *extremely relevant* for the first part, and from *strongly disagree* to *strongly agree* for the second part. We computed an individualizing (α = .62 for mothers, α = .69 for fathers) and a binding foundation score for each parent (α = .77 for mothers, α = .73 for fathers). Parents also completed short measures of socio-political ideology, parenting styles and social desirability; however, as these measures are not related to our main hypotheses, the results from these measures are not reported here. Their inclusion in the models that were tested did not alter the findings.

## Results

All the analyses were conducted using IBM SPSS Statistics Base (Version 24) and AMOS (Version 23) for the structural equation models (SEM). Descriptive statistics and correlations among all measures are displayed in Table 1. For each SEM aimed at assessing the link between parents’ and children’s responses, we ran a corresponding regression analysis in order to detect potential outliers based on Cooks’ distance [77]. Given the relatively small sample, we chose to be conservative and dropped only two cases with a very large Cook’s distance. They were a child-parent triad and a child-mother dyad with male children aged 9 and 10; in both cases, mothers reported very high levels of SDO. When relevant, for the sake of completeness, we also reported the findings from the analyses including these outliers.

**Table 1:** Descriptive statistics and correlations for children’s and parents’ measures.

		*M*	*SD*	2	3	4	5	6	7	8	9	10	11	12	13
1.	IAT	-.01	.49	.07	.09	.03	-.04	-.147	-.06	-.10	.00	.12	.01	-.02	-.14
2.	Behaviour evaluation	1.83	.88	--	**.49** ***	**.55** ***	**.51** ***	-.20*	.04	.11	-.10	**.24** **	**.20** *	.07	.04
3.	Intention to interact	1.58	.94		--	**.55** ***	**.43** ***	**-.27** **	.00	-.07	-.05	**.20** *	**.17** *	.00	.03
4.	Traits evaluation	1.34	.87			--	**.73** ***	-.21*	-.10	.11	**-.20** *	.15	.11	.00	-.08
5.	Traits comparison	3.78	2.07				--	-.13	.04	.04	-.10	**.22** *	**.18** *	.06	.07
6.	Mother’s SDO	1.81	.77					--	**.25** **	-.02	.14	**-.16**	.06	-.04	**.20** *
7.	Mother’s RWA	3.88	.78						--	-.07	**.64** ***	-.15	**.47** ***	-.02	**.50** ***
8.	Father’s SDO	3.41	1.10							--	-.07	-.04	-.03	.11	-.03
9.	Father’s RWA	4.04	.93								--	-.16	**.39** ***	-.14	**.51** ***
10.	Mother’s Individualizing	4.92	.48									--	**.33** ***	**.36** ***	.09
11.	Mother’s Binding	4.17	.64										--	.14	**.56** ***
12.	Father’s Individualizing	4.76	.58											--	**.30** ***
13.	Father’s Binding	4.23	.63												--

* *p* < .05

** *p* < .01

*** *p* < .001.

### Children’s attitudes

To test whether children showed a preference for the fair over the pro-ingroup protagonist, we ran a series of one-sample t-tests. Means were significantly greater than 0 for all measures, except for the implicit attitude score (*M* = -.01, *t* < 1), indicating a general preference, at the explicit level, for the fair over the pro-ingroup child: for the behaviour evaluation score, *t*(153) = 25.81, *p* < .001, 95% CI [1.70, 1.98], *d* = 2.07; for the intention to interact score, *t*(153) = 21.00, *p* < .001, 95% CI [1.44, 1.74], *d* = 1.69; for the traits evaluation score, *t*(153) = 19.12, *p* < .001, 95% CI [1.21, 1.49], *d* = 1.53; for the comparison along evaluative traits, *t*(153) = 22.82, *p* < .001, 95% CI [3.47, 4.13], *d* = 1.84. Because 95.5% of the respondents reported a preference for the fair child as a playmate and therefore there was virtually no variability in the responses, we did not further analyse this variable.

No gender differences were found for children’s measures, whereas age was negatively and significantly correlated with the traits evaluation score, *r*(152) = -.18, *p* = .027, suggesting that the preference for the fair over the pro-ingroup child attenuated with increasing age.

### Parental influences

To test whether parents’ responses were related to children’s inequality aversion, we separately tested a SEM for each parent. As for mothers’ responses, the model tested whether children’s explicit and implicit attitudes were predicted by mothers’ SDO and RWA mediated by their individualizing and binding foundations. The same SEM was run with the father’s responses. To control for inflated measurement errors due to multiple items for the latent variables, indicators were defined by parcelling: three item parcels were built for parents’ SDO and RWA, whereas the single foundations dimensions were used for parents’ individualizing (care and fairness) and binding foundations (ingroup, authority and purity). Turning to dependent variables, the indicators of children’s explicit attitude were behaviour evaluation, intention to interact, trait evaluation and trait comparison. For children’s implicit attitudes, the IAT score was used as a single indicator by specifying its regression coefficient and measurement error variance through Munck’s [78] formulae.

In line with Hu and Bentler’s suggestions [79], different indexes were combined to evaluate the fit of these models. Based on Schreiber et al. [80], the comparative fit index [CFI: 81], the Tucker–Lewis coefficient [TLI: 82], and the root mean square error of approximation [RMSEA: 83] were chosen. Based on Bentler [81] and Browne [84], the CFI and the TLI were considered as satisfactory if higher than 0.90. Moreover, based on Browne and Cudeck [85], the RMSEA was considered good if lower than 0.05 and acceptable if ranging between 0.05 and 0.08. Even if it was reported, the χ^2^ of the models was not taken into consideration, because such an index heavily depends on the N of the dataset.

#### Mediation models

As for the first mediation models, the one related to mothers showed an acceptable fit to the data: χ^2^ (91) = 138.90, *p* = .001, *CFI* = .93; *TLI* = .91, *RMSEA* = .06. Factor loadings were significant with *p* < .001 for all parcels and, when standardized, ranged between .57 and .96. Mothers’ RWA score significantly predicted their binding foundations, β = .67, *p* < .001. Although neither mothers’ SDO nor RWA scores had an indirect effect on children’s measures, *p*s > .20, their SDO score had a significant total effect on both children’s explicit, β = -.21, *p* = .032, 95% *CI* [-.40, -.01], *R^2^* = .12, *p* = .023, *f^2^* = .13, and implicit attitudes β = -.30, *p* = .012, 95% *CI* [-.60, -.08], *R^2^* = .18, *p* = .017, *f^2^* = .22.

The model related to fathers showed an even better fit: χ^2^ (91) = 111.56, *p* = .071, *CFI* = .97; *TLI* = .95, *RMSEA* = .04. Factor loadings were significant with *p* < .001 for all parcels (with an exception of *p* = .008) and, when standardized, ranged between 0.44 and 1.04. However, fathers’ measures did not significantly predict children’s measures and only their RWA score significantly predicted binding foundations, β = .79, *p* < .001. Both the indirect effects and the total effects of fathers’ SDO and RWA on children measures were not significant, *p*s > .37.

After these complete models, we tested two simpler models, separately for each parent, with either authoritarian attitudes or moral foundations. These models aimed to confirm the effect of mothers’ SDO and to check whether parents’ moral foundations still have no effects, even when authoritarian attitudes were not controlled for. In addition, to test whether the associations between parents’ and children’s measures were moderated by children’s age and gender, we subsequently added to each of the former models one of the moderators and its interaction with parents’ measures.

#### Parents’ authoritarian attitudes

**Fig 2:**
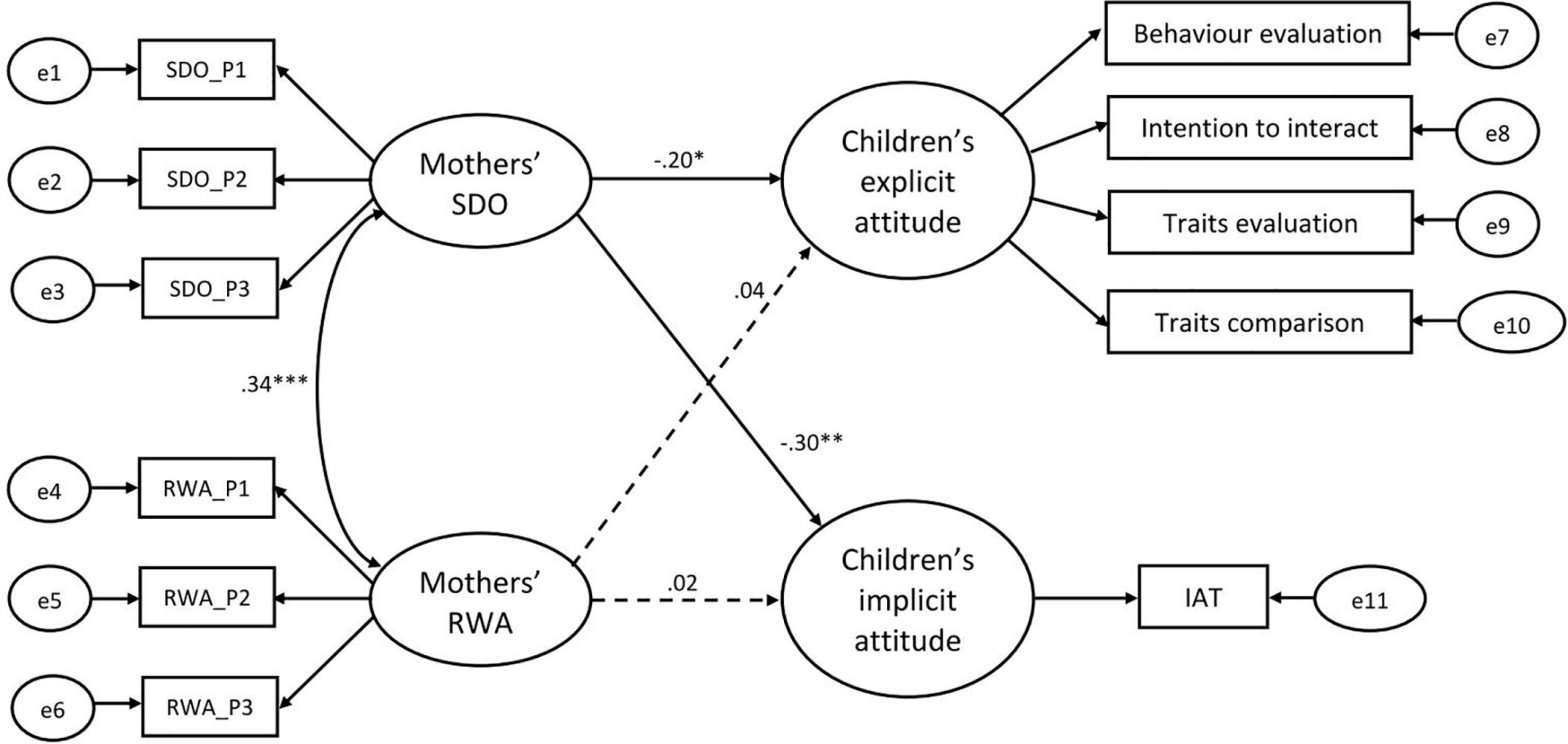
Mothers’ authoritarian attitudes model. *Note*: *Standardized beta coefficients were reported*. * *p* < .05, ** *p* < .01, *** *p* < .001.

As for the first models, the mothers’ one showed an acceptable fit to the data: χ^2^ (40) = 62.63, *p* = .013, *CFI* = .95; *TLI* = .94, *RMSEA* = .06. Factor loadings were significant with *p* < .001 for all parcels and, when standardized, ranged between 0.55 and 0.97. Whereas mothers’ RWA score did not significantly predict children’s measures, her SDO score predicted both children’s explicit, β = -.20, *p* = .044, 95% *CI* [-.42, -.01], *R^2^* = .04, *p* = .004, *f^2^* = .04 and implicit attitudes, β = -.30, *p* = .008, 95% *CI* [-.50, -.08], *R^2^* = .09, *p* = .002, *f^2^* = .09 (see Fig 2; when including the two outliers: β = -.13, *p* = .186, 95% *CI* [-.290, .040], β = -.23, *p* = .037, 95% *CI* [-.499, -.007], respectively). Importantly, in order to test the stability of the findings and that the exclusion of the two extreme outliers did not accidentally inflate Type I errors, we explored findings after the stepwise removal of other triads (up to 20) with the relatively highest Cook’s distance in our sample. At any further removal of a triad, findings did not substantially change. Indeed, the standardized β coefficient remained quite stable with values ranging between -.18 and -.23 in the case of explicit attitudes, and in the range between -.23 and -.33 in the case of implicit attitudes. This supports the conclusion that after the removal of the two more extreme outliers a consistent and stable pattern of finding emerges in relation to the predictive role of mothers’ SDO.

Fathers’ model showed an even better fit: χ^2^ (40) = 30.80, *p* = .852, *CFI* = 1.00; *TLI* = 1.03, *RMSEA* = .00. Factor loadings were significant with *p* < .001 for all parcels and, when standardized, ranged between 0.57 and 0.93. However, fathers’ measures did not significantly predict children’s measures.

Children’s gender did not moderate neither the effects of mothers’ SDO and RWA on children’s explicit and implicit attitude, χ^2^ (68) = 97.72, *p* = .011, *CFI* = .96; *TLI* = .94, *RMSEA* = .06, nor the effects of fathers’ SDO and RWA, χ^2^ (68) = 57.09, *p* = .840, *CFI* = 1.00; *TLI* = 1.02, *RMSEA* = .00. The same occurred for children’s age, but adding age and the two interaction terms worsened both mothers’ and fathers’ model fit, χ^2^ (68) = 134,24, *p* < .001, *CFI* = .88; *TLI* = .84, *RMSEA* = .08; χ^2^ (68) = 107.42, *p* < .001, *CFI* = .92; *TLI* = .89, *RMSEA* = .07, respectively.

#### Parents’ moral foundations

Mothers’ second model showed a good fit: χ^2^ (31) = 37.27, *p* = .203, *CFI* = .98; *TLI* = .97, *RMSEA* = .04. Factor loadings were significant with *p* < .001 for all foundations and, when standardized, ranged between 0.58 and 0.90. However, the paths from mothers’ to children’s measures were not significant. Fathers’ model showed a good fit: χ^2^ (31) = 33.43, *p* = .350, *CFI* = .99; *TLI* = .99, *RMSEA* = .02. Factor loadings were significant with p < .001 for all foundations except harm (*p* = .173) and, when standardized, ranged between 0.39 and 0.92. Fathers’ measures did not significantly predict children’s measures.

Children’s gender did not moderate the effects of neither mothers’ nor fathers’ individualizing and binding foundations, χ^2^ (56) = 70.78, *p* = .088, *CFI* = .97; *TLI* = .96, *RMSEA* = .04; χ^2^ (56) = 66.86, *p* = .152, *CFI* = .98; *TLI* = .97, *RMSEA* = .04 respectively. Likewise, children’s age did not moderate the associations between parents’ and children’s measures, χ^2^ (55) = 74.78, *p* = .039, *CFI* = .95; TLI = .93, *RMSEA* = .05 for mothers; χ^2^ (55) = 81.37, *p* = .012, *CFI* = .92; *TLI* = .89, *RMSEA* = .06 for fathers.

## Discussion

Findings clearly showed that children displayed an overall strong explicit preference for individuals behaving in a fair rather than unfair way. In line with previous research [e.g., 1, 2], children differentially evaluated the two targets, indicating that the embracement of a fairness norm was a key driving force. Critically, this pattern appeared even though the unfair behaviour actually favoured the ingroup. Hence, a norm towards egalitarianism emerged as far more influential than a pro-ingroup norm, at least on explicit measures. This effect did not emerge in relation to implicit attitudes. This discrepancy between the two types of measures could be attributed to measurement issues (i.e., a lower sensitivity of the IAT) or to the fact that indirect measures, being less influenced by social desirability concerns, might better capture the influence of pro-ingroup norms in this context. The first interpretation is less likely than the second, as mothers’ SDO score predicted children’s attitudes at both implicit and explicit level. In addition, consistently with the second interpretation, studies with adult populations have already shown that a preference for ingroup members who themselves favour the ingroup over the outgroup often emerges on implicit measures whereas on explicit measures a preference for egalitarian ingroup members is the most pervasive response [86, 87]. In a similar way, even children who, in the present study, explicitly expressed more positive attitudes towards the peer displaying fair behaviours, might have been overall more ambivalent in their spontaneous responses. This has been shown here for the first time and seems particularly interesting and worth of further exploration.

The major goal of the present research, however, was to explore how the relative impact of the two norms of fairness and ingroup favouritism among children could be modulated by parents’ social attitudes and moral standing. Indeed, the present study was primarily aimed to uncover whether the variability in children’s appraisal and reactions to unequal behaviours in intergroup settings is associated to their parents’ SDO, RWA, and moral foundations. Findings are largely consistent with those reported by Reifen Tagar and colleagues [43]. Importantly, the negligible impact of RWA was confirmed, whereas the orientation toward social dominance was found to be a major predictor of children’s responses as in Reifen Tagar et al. [43]. More specifically, it was here found that there was a significant path linking mothers’ SDO and children’s responses, whereas fathers’ SDO was not significantly associated to children’s evaluation of fair versus pro-ingroup targets. SDO has proved to be a largely stable disposition [88] that is a causal determinant of people’s intergroup attitudes [37, 88]. The present data suggest that SDO does not only shape attitudes at an intra-individual level, but it may also affect–at least in the case of mothers–the responses of one’s children when faced with intergroup discrimination. The stronger effect for mothers than fathers could reflect the fact that mothers often represent the primary caregivers and spend more time with children, thus having more chances to intervene in the socialization processes of children. However, this is only a tentative explanation and future studies will have to first assess the stability of this difference between mothers and fathers, and to uncover the variables behind the observed difference.

The present study extends previous work about the relevance of SDO [43], not only identifying the particular role of mothers, but also in several other respects. First, it was shown that the pattern emerged also in relation to meaningful social groups based on skin colour, and not just in the case of minimal groups. Second, the adopted experimental procedure helped to rule out alternative explanations based on a possible preference for individuals who end up having more resources. Indeed, in the present work the children distributing the resources could not keep them for themselves and therefore their behaviour could not be moved by self-interest but only by the desire to either be fair or favour another ingroup member. Third, mothers’ SDO predicted both explicit and implicit attitudes. Hence, there is a preliminary evidence that mothers can affect even less controlled responses, as those tapped by the child-friendly version of the IAT, that are likely to be less influenced by social desirability concerns. Knowledge about mothers’ SDO can thus represent an important element that enables to understand the more spontaneous responses of children when faced with discriminatory intergroup behaviours.

In an exploratory way, we have also assessed the potential role of moral intuitions. Indeed, parents’ individualizing foundations, comprising an attention to care and fairness, could be expected to be positively linked to children’s inequality aversion. In a similar way, parents’ binding foundations, pointing to the value associated to ingroup loyalty, could be expected to be linked to children’s increased preference for pro-ingroup behaviours. Our findings, however, did not support these predictions. More specifically, the simultaneous inclusion of SDO and moral foundations in the model showed that the latter factors did not uniquely contribute to predict children’s responses. From a theoretical point of view, the observed pattern appears to be more in line with a motivated social cognition account over a perspective that gives primacy to moral intuitions [64, 65].

### Limitations and future directions

Although in the present study parents’ RWA was not significantly associated with children’s responses, it might be premature to conclude that RWA is always uninfluential. Indeed, in the adopted experimental procedure, the strongly unequal distribution of resources to the two targets (i.e., 8 versus 2) might have made more prominent its extreme unfairness at the expenses of its connection to ingroup favouritism. Future studies could thus manipulate the absolute relevance of the unfair behavior and the salience of intergroup differentiation, and stress that the pro-ingroup distribution choices were indeed motivated by a desire to favour the ingroup. In this way, the tension between the two conflicting norms of egalitarianism and loyalty towards the ingroup would be further accentuated. Similarly, an increased salience of the threatening nature of the outgroup could result in an increased relevance of authoritarianism [36, 62].

In the present work, mothers’ and children’s attitudes towards the specific outgroup (i.e., Black people) were not assessed, but future studies would benefit from the inclusion of such measures as possible mediators of the observed effects of SDO. In other words, mothers’ SDO, through their intergroup attitudes, might significantly influence children’s attitudes which, in turn, might shape their reactions to unequal behaviours that favour the ingroup while damaging the outgroup.

Another important limitation of the present work is its cross-sectional nature, strongly pointing to the need of longitudinal studies in order to more directly investigate causal effects, also including a larger sample to more properly address eventual age-related differences. In addition, as we focused on a western, educated, industrialized, rich and democratic (WEIRD [89]) sample, further studies could explore whether a different pattern of finding would emerge in more collectivist and traditional cultures.

Notwithstanding these drawbacks, the current study extends in several ways the so far limited knowledge about the possible role of parents in shaping children’s reactions to inequality in intergroup contexts. Overall, results confirmed previous research findings indicating children’s inequality aversion at the explicit level, although the same strong embracement of this fairness norm did not emerge at the implicit level. More importantly for the main goals of the present study, results consistently point to the relevance of mothers’ SDO–and the negligible role of both RWA and moral foundations—in relation to the way in which children balance the two competing drives of equality endorsement and pro-ingroup bias. The present findings thus further stress the significance of social dominance as a key construct for understanding not only how we perceive our social world but also how our views about such a world are perpetrated and transmitted to the next generations.
